# Two cysteines control Tse1 secretion by H1‐T6SS in *Pseudomonas aeruginosa*

**DOI:** 10.1002/pro.70226

**Published:** 2025-07-28

**Authors:** Marie M. Grandjean, Jean‐Pierre Duneau, Edwige B. Garcin, Laetitia Houot, Olivier Bornet, Christophe Bordi, Latifa Elantak, Corinne Sebban‐Kreuzer

**Affiliations:** ^1^ Aix‐Marseille Univ., CNRS, LISM UMR7255, IMM FR3479, Laboratoire d'Ingénierie des Systèmes Macromoléculaires Institut de Microbiologie de la Méditerranée Marseille France

**Keywords:** amidase, cysteine residues, disulfide bond, H1‐T6SS, NMR, *Pseudomonas aeruginosa*, redox protein, Tse1

## Abstract

Type Six Secretion Systems (T6SS) are molecular machines that export toxic effector proteins into bacterial competitors or eukaryotic cells. *Pseudomonas aeruginosa*'s H1‐T6SS secretes Tse1, which contains a disulfide bond between cysteines at positions 7 and 148, linking its N‐ and C‐terminal regions. The role of this disulfide bond in Tse1 activity and mechanism of action during bacterial competition is unknown. In this study, we investigated the role of the C7‐C148 disulfide bond within Tse1. First, NMR spectroscopy experiments suggest a redox‐active instead of a structural disulfide bond. Moreover, while the presence of this bond did not alter Tse1's amidase activity or toxicity in *Escherichia coli*, substituting cysteines C7 or C148 in *P. aeruginosa* strains affected the bacterium's capacity to lyse prey cells. Secretome analysis showed that the Tse1C148S variant was not secreted via the H1‐T6SS, whereas the Tse1C7S variant was secreted. These findings suggest that cysteine 148 is likely important for Tse1's assembly with the T6SS machinery, while cysteine 7 appears to be involved in its disassembly, potentially through the formation of the disulfide bond. This study points to a potential redox regulation mechanism during the assembly and disassembly of Tse1 with Hcp1, consistent with a “bridge of delivery” model.

## 1 INTRODUCTION

Disulfide bonds have classically been shown to stabilize proteins by maintaining protein overall structure via intermolecular and intra‐domain covalent bonds between two cysteine residues. These structural disulfide bonds are essential for the stability of secreted proteins destined for the harsh oxidizing extracellular environment, but also in the regulation of proteins function. In gram‐negative bacteria, each cell compartment is defined by a specific redox potential: low in the cytoplasm (~−230 mV), which turns it into a reducing environment, and higher in the periplasm (~−165 mV) which allows the oxidative folding of proteins with disulfide bonds formation (Carmel‐Harel and Storz 2000; Denoncin et al. 2013).

Bacterial secretion systems, such as the SEC or TAT translocons and the type I to XI secretion systems, enable the translocation of compartment‐specific proteins within bacterial cells, the surrounding environment, or neighboring cells, whether they are hosts or competitors (Green and Mecsas 2016). These translocated proteins must contend with significant changes in the redox potential of their environment.

Type Six Secretion Systems (T6SSs) are contact‐dependent, phage‐like contractile nanoweapons implicated mainly in bacterial competition. They facilitate the translocation of toxic effectors from the cytoplasm of a predatory cell into a prey cell (Granato et al. 2019; Hood et al. 2010). As recently reviewed, effector activities are diverse and can target periplasmic components (cell walls, membranes) or cytoplasmic components, including nucleic acids and essential metabolites (Hernandez et al. 2020; Jana and Salomon 2019; Jurėnas and Journet 2021). These T6SSs are highly conserved in Proteobacteria and are composed of a minimum of 14 different proteins organized into three multimeric components: a membrane complex (TssJLM), a baseplate structure composed of six wedges assembled from proteins TssAEFGK that surround the VgrG‐PAAR spike complex, and a tube‐sheath complex assembled by Hcp and TssBC proteins (Cascales and Cambillau 2012; Cianfanelli et al. 2016; Nguyen et al. 2018; Zoued et al. 2014).

The inner tube of the T6SS apparatus is composed of hexamers of the Hcp protein, capped by a spike complex formed by a trimer of the VgrG protein and sharpened by a PAAR protein. This tube serves as a conduit for effectors, which are propelled into the target cell upon contraction of the TssBC sheath. Some effectors, known as cargo effectors, assemble inside the Hcp tube through non‐covalent interactions, although no conserved secretion signal shared by all Hcp‐associated effectors has yet been identified. It remains unclear whether these effectors are recruited in a folded or unfolded state. Hcp plays a dual role, serving both as the conduit and as a chaperone‐like stabilizer for certain effectors (Silverman et al. 2013). The mechanisms governing the release of effectors from the Hcp tube after secretion into the target cell remain poorly understood.

*Pseudomonas aeruginosa*, a gram‐negative bacterium, is responsible for both acute and chronic infections in humans. Acute infections are characterized by toxin production, whereas chronic infections involve biofilm formation, where bacteria utilize the T6SS for host cell invasion (Furukawa et al. 2006; Gooderham and Hancock 2009). *Pseudomonas aeruginosa* harbors three main type VI secretion systems, designated H1‐, H2‐, and H3‐T6SSs (Mougous et al. 2006). A recent study, however, has identified a fourth system (H4‐T6SS) present in approximately 1% of *P. aeruginosa* genomes (Habich et al.  2025). The H1‐T6SS was the first identified T6SS with documented antibacterial activity (Hachani et al. 2013; Hood et al. 2010; Mougous et al. 2007; Pissaridou et al. 2018; Russell et al. 2011). The H1‐T6SS is tightly regulated (Brencic et al. 2009; Brencic and Lory 2009; Casabona et al. 2013; Goodman et al. 2004; Hsu et al. 2009; Mougous et al. 2007) and has been described as a defensive system (Basler et al. 2013; Ho et al. 2013). The H1‐T6SS of *P. aeruginosa* PAO1 strain deploys at least eight effectors (Tse1‐8) with synergistic antibacterial activities (LaCourse et al. 2018; Nolan et al. 2021). These effectors target a range of prokaryotic structures, including the cell wall peptidoglycan, cytoplasmic membrane, NAD(P)+, DNA, and the transamidosome. Tse1 and Tse3 act on the peptidoglycan, Tse4 and Tse5 target the cytoplasmic membrane and exhibit bactericidal or bacteriostatic activities, while Tse2, Tse6, Tse7, and Tse8 are translocated into the prey's cytoplasm to exert diverse toxic activities (Chou et al. 2012; González‐Magaña et al. 2020; Hood et al. 2010; LaCourse et al. 2018; Li et al. 2013a; Lu et al. 2014; Nolan et al. 2021; Pissaridou et al. 2018; Radkov et al. 2022; Russell et al. 2011; Whitney et al. 2014; Whitney et al. 2015). This diverse range of effectors highlights the sophisticated role of the H1‐T6SS as a defense system in *P. aeruginosa*.

Interestingly, Tse1‐4 Hcp1‐cargo effectors do not appear to share a common motif for interaction with Hcp1. Instead, each effector exhibits a specific interaction pattern within the lumen of Hcp1 (Silverman et al. 2013), raising questions about the mechanism(s) of effector recruitment within Hcp1 hexamers.

Tse1 is a cysteine protease featuring a catalytic Cys‐His diad and comprises six cysteine residues within its sequence. It functions as an amidase, cleaving g‐D‐glutamyl‐meso‐2,6‐diaminopimelic acid (D‐Glu–mDAP) bonds and collaborates with the muramidase Tse3 to degrade the prey's envelope (Chou et al. 2012; Lu et al. 2014; Radkov et al. 2022; Russell et al. 2011). While the crystal structures of Tse1 reveal a protein comprising a three‐stranded central β‐sheet and five α‐helices arranged as α1‐α2‐α3‐α4‐β1‐β2‐α5‐β3, the presence of a disulfide bond between C7 and C148 cysteines, connecting the protein's N‐terminal and C‐terminal ends, is unlikely in the reducing cytoplasm of *P. aeruginosa* (Benz et al. 2012; Chou et al. 2012; Ding et al. 2012; Shang et al. 2012; Zhang et al. 2012). Tse1 is secreted into the periplasmic space of the prey bacterium, an oxidizing environment that promotes disulfide bond formation, which could help stabilize its native conformation or support efficient enzymatic activity.

In this study, we aimed to investigate the role of the C7 and C148 cysteines in Tse1. Using NMR spectroscopy and in vitro amidase activity assays in both redox states, we showed that these cysteines did not affect activity but rather influenced the structural properties of the N‐ and C‐terminal regions of the enzyme. To investigate the role of these cysteines during the Tse1 export process from the cytoplasm of *Pseudomonas aeruginosa* to the periplasm of a target cell, we examined the impact of substituting these residues in various *Pseudomonas* strains through secretome analysis and bacterial competition assays. Our findings provide evidence suggesting redox regulation in the assembly and disassembly of the cargo effector with the Hcp1 protein.

## 2 RESULTS

### 2.1 The C7‐C148 disulfide bond modulates Tse1 structure

**FIGURE 1:**
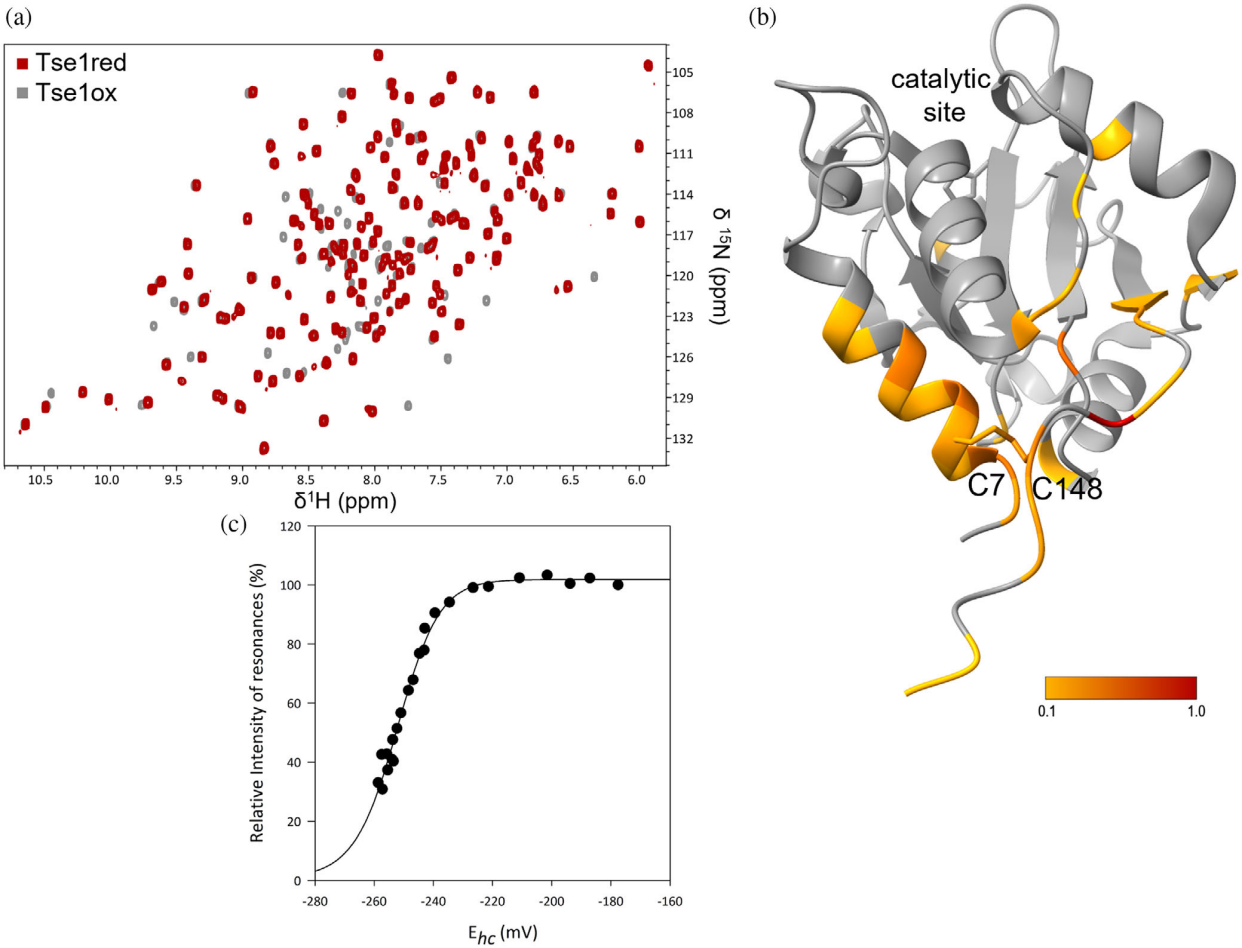
Influence of the C7‐C148 disulfide bond on Tse1 structure. (a) Overlay of ^1^H,^15^N‐HSQC spectra of Tse1 in its reduced (red) and oxidized (gray) states to 1 mM, acquired at 300 K on a Bruker Avance III 600 MHz spectrometer, in 100 mM NaCl, 10 mM KPO_4_ buffer at pH 7, with and without 10 mM DTT, and 10% D_2_O. (b) Chemical shift deviations (CSDs) for resonances between reduced and oxidized Tse1 states, are displayed on the oxidized Tse1 structure (PDB ID: 4FOV) (Shang et al. 2012). (c) Redox potential of the Tse1 disulfide bond: changes in NMR signal intensities for NH resonance of Tse1‐C148 under oxidizing and reducing conditions plotted against the half‐cell potential of glutathione. Black circles represent signal intensity in the oxidized state. Redox potentials were determined using the Nernst equation based on the ratio of reduced and oxidized glutathione concentrations. Experimental data were fitted using this same function.

To study the role of the disulfide bond in Tse1, we conducted a structural analysis using NMR (Figure 1a). Here, we present the backbone resonance assignments for Tse1 in both redox states (Figure S1, Supporting Information). While the assignments for the oxidized form were previously reported by Radkov et al. (2022), we assigned resonances of the reduced form and performed an analysis of the chemical shift differences between the two redox states for each NH resonance (Figure S2). The obtained values were then mapped onto the Tse1 structure (Figure 1b). These data reveal a significant variation in chemical shift values between the two redox states, notably affecting the entire α1 helix and the C‐terminal loop extending from the β3 strand. This analysis highlights that the absence of the disulfide bond destabilizes the α1 helix positioning, likely resulting in a more open and flexible conformation in the reduced form of Tse1.

The nature of a disulfide bond, whether structural or redox‐active, can be determined by measuring its redox potential. Structural disulfide bonds typically exhibit very low redox potentials, which can extend to −470 mV (Wouters et al. 2010). In contrast, redox potentials for disulfides in active sites, such as those found in thiol–disulfide oxidoreductases, or for allosteric disulfides, generally range from −120 to −270 mV (Li et al. 2015). We defined the redox potential of Tse1 through ^1^H‐^15^N HSQC NMR experiments, using the redox couple of reduced (GSH) and oxidized (GSSG) glutathione as references (Garcin et al. 2012; Piotukh et al. 2007). The intensities of the NH resonances of the oxidized form of Tse1 decreased upon the addition of reduced glutathione, while the intensities of the NH resonances of the reduced form increased. Analyzing the NMR signal intensities of NH resonances yielded a curve that was successfully fitted using the Nernst equation to determine the redox potential (Figure 1c). For the cysteines involved in the disulfide bridge of Tse1, the redox potential determined was −253 ± 3.7 mV. This measurement suggests that the disulfide bond in Tse1 is likely redox‐active rather than purely structural, implying a functional role in redox processes under oxidizing conditions.

To mimic in vivo Tse1 reduced conformation, we produced variants in which cysteines were substituted with serines to minimize chemical disturbance. These variants were produced and analyzed using NMR (Figure S3). The analysis of chemical shift deviations (CSDs) between the variants and the reduced form of Tse1 showed minimal variations, particularly within the α1 helix for both variants and slightly in the C‐terminal region for Tse1C148S. This can be attributed to the intrinsic disorder in the last 13 residues of Tse1's C‐terminal region.

### 2.2 The C7‐C148 disulfide bond does not influence Tse1 amidase activity

**FIGURE 2:**
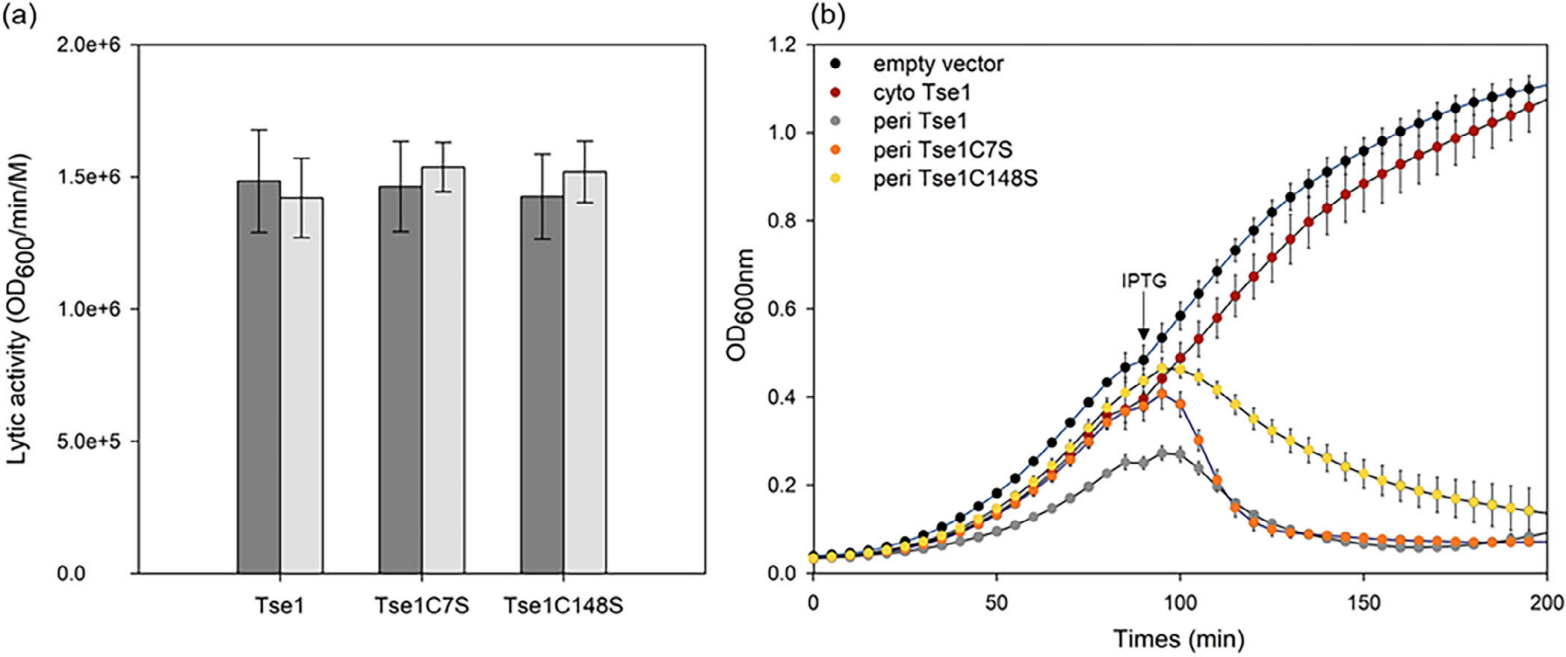
Influence of the C7‐C148 disulfide bond on Tse1 amidase activity. (a) In vitro amidase activity of Tse1 was evaluated using permeabilized *E. coli* TG1 strain. Permeabilization of *E. coli* TG1 was achieved by using TES buffer (40 mM Tris–HCl, pH 8.0, 0.5 μM EDTA, 0.5M sucrose). Tse1 and its variants were assayed at a final concentration of 20 nM, with and without 10 mM DTT, using 1 mL of permeabilized bacterial mixture. Each condition was tested in five independent replicates. Statistical analysis was performed using SigmaPlot: unpaired Student's *t* tests were applied to compare different enzymes, and paired *t* tests were used to compare the same enzyme with and without DTT. Hydrolysis rates are presented as mean ± SD. No statistically significant differences were observed (all *p*‐values >0.05; lowest *p* = 0.176). (b) Growth of *E. coli* EAEC 17.2 carrying pMMB67HE plasmids for IPTG‐inducible expression of the listed proteins fused with or without an N‐terminal PelB signal peptide. Cultures were grown in LB ampicillin, and growth at 600 nm was monitored using a Tecan instrument. *E. coli* strains expressing Tse1 in the cytoplasm or carrying the empty vector served as controls. An arrow marks the timepoint (90 min) when IPTG (1 mM) was added to the media. Data are presented as mean ± SD; *n* = 3 biological samples. The experiment was independently repeated four times with consistent results.

We performed in vitro amidase activity assays using purified Tse1, Tse1C7S, and Tse1C148S on permeabilized *Escherichia coli* cells. As shown in Figure 2a, the in vitro lytic activity of Tse1 was determined to be at 1.48.10^5^ ± 0.19 OD_600_/min/M, consistent with previously published data (Chou et al. 2012). Remarkably, the presence of DTT in the buffer did not affect Tse1 activity and the substitution of the two cysteines also had no impact on the toxin's lytic activity. These results suggest that the redox state of Tse1 does not influence its catalytic activity.

Next, we assessed Tse1‐dependent toxicity in the periplasm of, *E. coli* BL21, EAEC 17.2, and in *Pseudomonas aeruginosa Δtsi1Δtse1* strains. Tse1, Tse1C7S, and Tse1C148S were expressed in the periplasm using an inducible plasmid which produces proteins fused to the PelB signal peptide, and the bacterial growth was monitored. In all cases, the results were very similar. Upon induction, cytoplasmic Tse1 had no effect on bacterial growth. In contrast, both the wild‐type Tse1 and cysteine variants exhibited clear toxicity when expressed in the periplasm (Figures 2b and S4). Although the variants displayed slightly reduced toxicity compared to the wild type, this is likely due to lower expression levels rather than a significant loss of lytic activity. In *P. aeruginosa*, the induction led to cell lysis without a decrease in OD, instead reaching a plateau with increased standard deviation, caused by cell debris precipitation interfering with Tecan measurements. These findings collectively suggest that the N‐ and C‐terminal cysteines of Tse1 have minimal to no impact on its amidase activity, regardless of the genetic background. Therefore, these results refute the hypothesis that the formation of a C7‐C148 disulfide bond could have been necessary to activate the enzyme in the periplasm of the prey cell.

### 2.3 Cysteines C7 and C148 in Tse1 are crucial for H1‐T6SS‐mediated prey lysis

**FIGURE 3:**
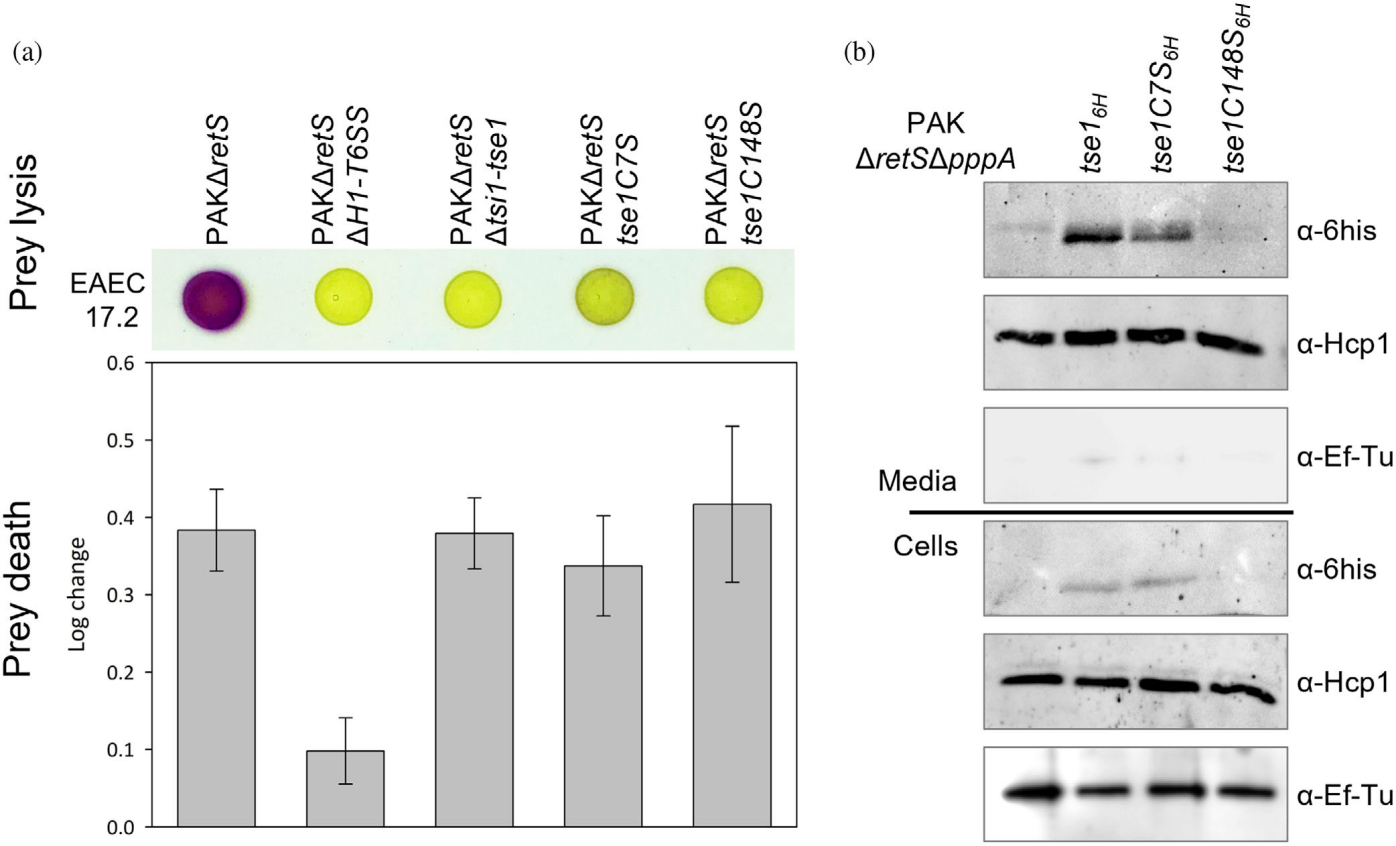
Tse1‐mediated prey lysis and secretion assays in *Pseudomonas*. (a) Tse1‐mediated prey lysis in bacterial competition assays. Prey lysis (top) and death (bottom) of *E. coli* EAEC 17.2 LacZ+ were assessed following co‐culture with various PAKΔ*retS* predator strains. (top) The addition of CPRG (a yellow beta‐galactosidase substrate) directly onto co‐culture spots reveals prey lysis via beta‐galactosidase activity, indicated by the production of purple CPR in the extracellular medium. (bottom) *E. coli* prey cells were grown with gentamycin on a microplate reader after co‐culture, and the time of emergence in the growth curve was determined and compared to a standard curve with serial dilutions of the prey cultured alone to obtain a log value. The log change values represent the number of prey deaths relative to the control (prey cultured alone) and are presented as mean ± SD. Only the PAKΔ*retS*Δ*H1‐T6SS* strain shows statistically significant differences compared to all other strains (*p* < 0.0001). (b) Tse1 secretion assays. Protein secretion was visualized using western blots on different PAKΔ*retS*Δ*pppA* strains. Tse1 was his‐tagged on the chromosome to track its secretion through western blot analysis of culture supernatants and its expression in the cell pellets. The first lane of each western blot serves as the control with no tag on Tse1. EF‐Tu was used as a sample loading control, and Hcp1 was used to control the functioning of the H1‐T6SS machinery.

Given that Tse1 plays a role in H1‐T6SS‐mediated killing of bacterial competitors, we aimed to investigate whether the absence of the disulfide bond between the N‐ and C‐terminal cysteines of the amidase would impact the killing of an *E. coli* prey by *P. aeruginosa*. To do so, we generated two mutants, *tse1C7S* and *tse1C148S*, at the chromosome locus in different *P. aeruginosa* strains. To observe H1‐T6SS‐mediated killing, we utilized a PAKΔ*retS* background strain in which the H1‐T6SS is expressed and functional (Brencic et al. 2009; Goodman et al. 2004). Since H1‐T6SS serves as a defensive system (Basler et al. 2013), we used an aggressive T6SS‐active *E. coli* strain, EAEC 17.2, as prey to activate H1‐T6SS‐mediated killing. As a negative control for specific H1‐T6SS killing activity, we constructed a PAKΔ*retS*ΔH1‐T6SS strain, as previously described (Hachani et al. 2013). We also generated a PAKΔ*retS*Δ*tsi1*‐*tse1* strain with toxin deletion to specifically assess the effect of Tse1 in our experiments. Because Tse1 has been reported to promote prey cell lysis without affecting prey cell death, which is mediated by other T6SS effectors (Chou et al. 2012; Rudzite et al. 2023; Russell et al. 2011), we used two complementary methods to assess bacterial prey lysis and bacterial prey death following co‐culture (Taillefer et al. 2023). The first method, known as the lysis‐associated β‐galactosidase assay (LAGA), relies on the measurement of β‐galactosidase activity released into the media after prey cell lysis. This method enables the assessment of bacterial prey lysis by monitoring the hydrolysis of a yellow, cell‐impermeable substrate (CPRG) into a dark pink product (CPR) (Figure 3a, top). The second method, termed survivors growth kinetics (SGK), is a quantitative approach used to evaluate bacterial cell death (in log scale). This is achieved by measuring survivor prey growth recovery using a microplate reader (Figure 3a, bottom). As expected, the PAKΔ*retS* predator induces prey lysis and death after co‐culture, whereas the PAKΔ*retS*ΔH1‐T6SS predator lacks this capability. Similarly, the PAKΔ*retS*Δ*tsi1*‐*tse1* strain is unable to lyse the prey, though the absence of Tse1 does not impact prey death. Interestingly, it is noteworthy that in the absence of Tse1, *Pseudomonas* loses its ability to lyse its prey, despite the presence of effectors Tse3, Tse4, and Tse5 (González‐Magaña et al. 2022; Le et al. 2021; Lu et al. 2014). This observation may be attributed to different experimental conditions, as Tse3 requires Ca^2+^ for its cell lysis activity (Li et al. 2013b), and, importantly, to the synergistic effects previously observed between Tse1 and Tse3 (Goodman et al. 2004), as well as to the fact that Tse4 and Tse5 alone do not induce bacterial lysis but rather form ion‐selective pores (Rojas‐Palomino et al. 2025a; Rojas‐Palomino et al. 2025b).

The substitutions C7S and C148S in Tse1 have a surprising and significant impact on prey lysis but not on prey death. We also observed that these two substitutions do not have an identical effect on prey lysis. The C7S mutant still generates a slight purple coloration after co‐culture, indicating some degree of prey lysis. However, the dramatic change in prey lysis observed during the competition assay cannot be attributed to a loss of amidase activity or instability of the mutants, as demonstrated above.

### 2.4 Cysteines C7 and C148 differentially regulate Tse1 secretion through the H1‐T6SS

To elucidate the essential role of the Tse1 disulfide bond in vivo, we next monitored the secretion of Tse1, Tse1C7S, and Tse1C148S. While most described T6SS are offensive systems, meaning that the assembly–secretion–disassembly cycle is constitutive, the H1‐T6SS is a defensive system where this cycle is post‐translationally regulated to counter‐attack after the aggression of another adjacent cell (Hsu et al. 2009; Mougous et al. 2007). It is then possible to constitutively activate the secretion of H1‐T6SS effector in strains lacking pppA, which is the major repressor of this pathway (Mougous et al. 2007).

We engineered a PAK*ΔretSΔpppA* strain in which Tse1 was chromosomally tagged with a His‐tag to facilitate its monitoring via western blot in culture supernatants, enabling assessment of secretion, expression, and stability (Figure 3b). Secretome and cellular content analyses were conducted on samples collected at mid‐log phase. EF‐Tu was used as a loading control, while Hcp1 was monitored to ensure proper assembly and function of the H1‐T6SS machinery. As expected, Tse1 was detected in both the cells and secretome of the PAK*ΔretSΔpppA* strain. Similarly, the C7S variant was present in both the cellular and secreted fractions. However, Tse1C148S was barely detectable in both the culture supernatant and the cells. Hcp1 secretion remained unaffected in these mutants, indicating no inherent defect in the H1‐T6SS secretion machinery. Importantly, our results reveal distinct secretion phenotypes for the two Tse1 variants. While Tse1C148S is not secreted, we do not have direct evidence of an interaction defect with Hcp1. Nevertheless, its absence from the pellet fraction may reflect impaired recognition, recruitment, or stabilization by Hcp1 in the cytoplasm. Tse1C7S is secreted correctly, although at a slightly lower level than Tse1, but it does not induce bacterial prey lysis. These findings suggest that the two cysteines play different roles in mediating bacterial prey lysis by Tse1.

### 2.5 The C7S substitution reduced Tse1 orientation variability in the Hcp1 ring

**FIGURE 4:**
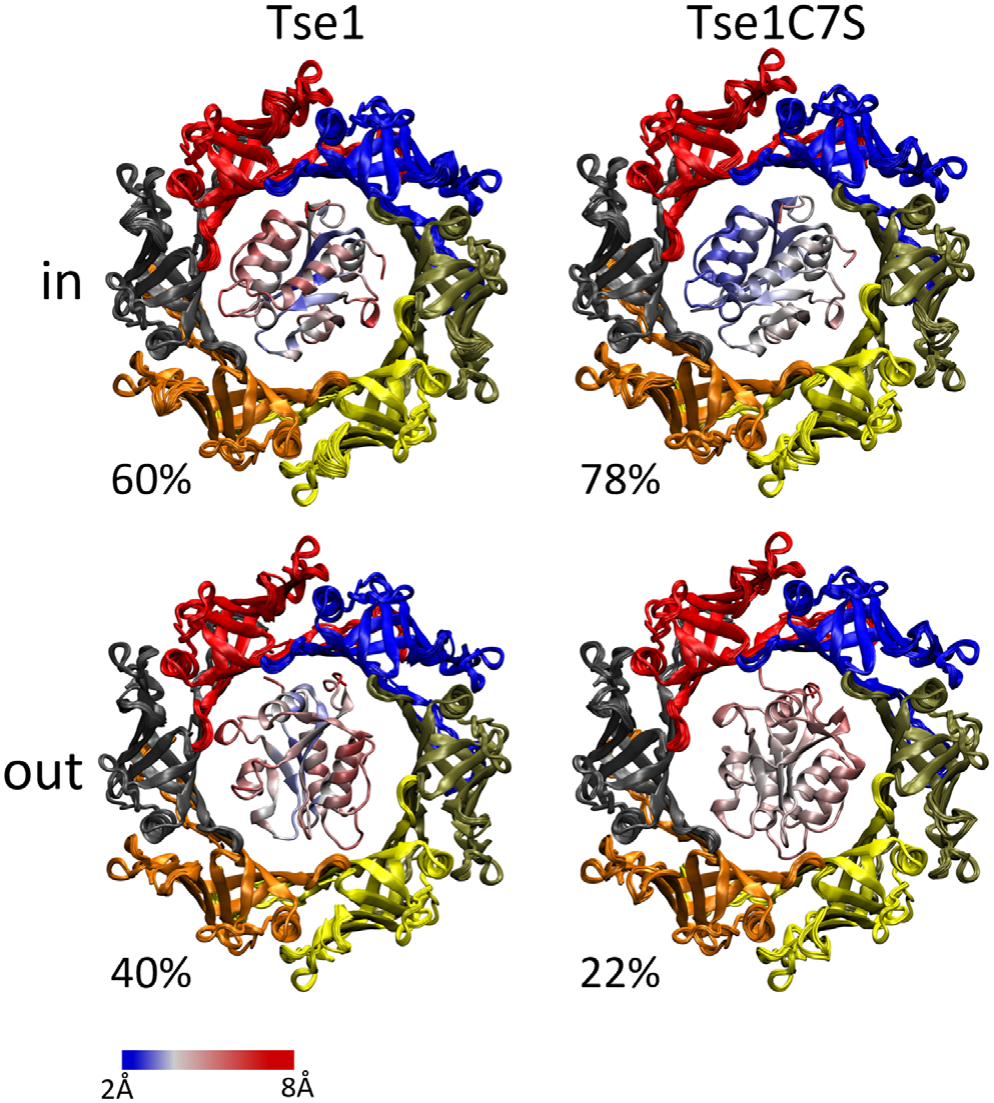
Variability of Tse1 position in the Hcp AlphaFold complex. Forty (Tse1, left) and forty‐six (C7S, right) AlphaFold predictions were conducted on Hcp‐Tse1 complexes. Variations in the total number of models reflect the exclusion of predicted assemblies that did not match known Hcp ring structures (e.g., pentamers or helicoidal forms). The Hcp ring is depicted from its cytoplasmic‐facing side when assembled in the T6SS secretion system. The panels display the superposition of the rings, color‐coded according to their assigned subunits. The A subunit (yellow) corresponds to the unit that interacts most with Tse1. The subsequent subunits (orange, black, red, blue, and tan) are arranged in a clockwise manner starting from the A position (see methods). The “in” and “out” positions indicate the relative position of Tse1 with respect to this Hcp reference face, with “in” representing the WT Tse1 disulfide bond facing the cytoplasm and “out” indicating it faces the exit edge of the secretion apparatus. The percentages refer to the proportion of valid Hcp:Tse1 ring models adopting each orientation. For example, in the case of wild‐type Tse1, 16 out of 40 (40%). The Tse1 proteins are represented within the ring and color‐coded by the standard deviation measured for each atom across the various models (from blue <2 Å to red >8 Å).

To understand the phenotypic differences between Tse1 and its C7S variant, structural modeling of each protein within the Hcp1 hexamer was performed using AlphaFold3 (Abramson et al. 2024) (Figure 4). All models generated for Tse1 depict the toxin in its oxidized form, simulating the complex in an oxidative environment such as the periplasm of the target bacterium during the disassembly stage.

The predicted structures of Tse1 and Tse1C7S are highly similar, with backbone RMSD values of 1.09 Å ± 0.52 for Tse1 and 0.92 Å ± 0.43 for the C7S variant. However, when examining the RMSD of the Tse1 proteins within the context of the fitted Hcp rings, we observed notable variations in Tse1 positioning. Both the wild‐type and C7S variant predominantly adopt an orientation in which the cysteines of the toxin point toward the cytoplasm (referred to as the “in” orientation). Interestingly, the positioning of the wild‐type protein appears less consistent compared to the variant. Specifically, the “out” orientation, accounting for 40% of the interaction modes in the wild‐type, is observed in only 22% of cases for the variant. Analysis of the standard deviation (depicted as a blue to red color gradient) of the Tse1 atomic positions in the AlphaFold models revealed greater variability in the orientation of oxidized Tse1 compared to the C7S variant (Table S1). This reduced variability in the orientation of the C7S variant suggests that the cysteine substitution likely stabilizes the effector's interaction with the Hcp1 ring, potentially preventing its disassembly.

## 3 DISCUSSION

In this study, we elucidated the roles of cysteines 7 and 148 in Tse1 function in *Pseudomonas aeruginosa*. While these residues do not directly contribute to peptidoglycan hydrolysis, as confirmed by in vitro amidase activity assays and toxicity assays in *E. coli*, their substitutions resulted in either complete or partial abolition of prey lysis during bacterial competition. Our findings show that the C‐terminal cysteine, C148, is essential for Tse1 secretion via the H1‐T6SS machinery, which explains the observed loss of prey lysis.

Proper secretion of cargo effectors through the H1‐T6SS apparatus relies on their correct assembly within the secretion machinery. Notably, we detected only minimal amounts of Tse1C148S in the cell pellets compared to the wild‐type and Tse1C7S variants, suggesting that the Tse1C148S variant exhibits reduced stability in the *P. aeruginosa* cytoplasm.

Hcp1 plays a dual role in *P. aeruginosa*, recruiting effectors for secretion and stabilizing them in the cytoplasm through a chaperone‐like activity (Silverman et al. 2013). This dual functionality suggests that effectors are either susceptible to cytoplasmic proteases or remain partially folded in the cytoplasm. Hcp1 has been shown to act as a chaperone for effectors such as Tse1, Tse2, Tse3, and Tse4, ensuring their stability. Similar mechanisms of degradation or reduced production have been reported for other effectors in various secretion systems, such as substrates of the Tat machinery (Lindenstrauss et al. 2010).

Although Tse1C148S is correctly expressed in a heterologous host such as *E. coli*, this variant appears to have impaired interactions with the hexameric protein Hcp1 in vivo. Its limited recognition and processing by the H1‐T6SS machinery in *P. aeruginosa* may have contributed to its destabilization and impaired secretion. These findings highlight the essential role of cysteine 148 in facilitating the correct association of Tse1 with Hcp1, a prerequisite for its stability and secretion.

Concerning the N‐terminal cysteine C7, it is not directly involved in Tse1 secretion through the H1‐T6SS. However, the significant reduction in prey lysis observed in the PAKΔ*retStse1C7S* during bacterial competition raises an intriguing question: how could a toxin with intact amidase activity and functional secretion fail to lyse a bacterial prey? While our secretion experiments do not directly track the fate of Tse1 after translocation into the prey cell periplasm, we hypothesize that the observed loss of prey lysis in the PAKΔ*retStse1C7S* mutant may be linked to a defect in a crucial event required for Tse1 toxicity in the prey's periplasm. Moreover, since substituting cysteine 7 with a serine does not impair Tse1 secretion, these results also suggest that the disulfide bond between the two cysteines is not essential for the protein secretion through the apparatus. In the oxidative environment of the prey's periplasm, disulfide bond formation is catalyzed by thiol‐disulfide oxidoreductases (Denoncin et al. 2013). After translocation into the prey's periplasm, Tse1 is likely oxidized due to the redox potential of this compartment, with the N‐ and C‐terminal cysteines expected to form a disulfide bond. In this context, we believe that in the Tse1C7S variant, the absence of disulfide bond formation could potentially disrupt the disassembly of the cargo effector with Hcp1, thereby explaining the loss of prey lysis by the PAKΔ*retStse1C7S* strain. This hypothesis is supported by the structural modeling analysis, which shows lower variability for the C7S variant in the Hcp1 ring compared to the oxidized form of Tse1. This highlights the intricate role of cysteine residues and disulfide bond dynamics in modulating the function and toxicity of T6SS effectors within bacterial competition scenarios.

A comprehensive analysis of the role played by individual amino acids within Tse1 was conducted by Radkov et al. (2022). Their study employed a sophisticated high‐throughput genetic approach, deep mutational scanning (DMS) (Furchtgott et al. 2011), to explore the entire Tse1 enzyme for functional determinants of toxicity in *E. coli*. This extensive analysis identified several key amino acids critical for amidase binding to peptidoglycan, specifically Leu21, Gly42, Gly48, Ala52, Gly56, and Ser144. However, their study did not identify any role for residues C7 and C148. It is important to note, though, that their method primarily focused on amino acids involved in catalysis, such as substrate binding and catalytic steps in which residues C7 and C148 are not implicated. Instead, their role is linked to the delivery of the toxin to the prey, a step not covered by their approach. This highlights the importance of every step in the toxin's mode of action: from its folding within the cell, through its delivery to the target cell, to the activation of its toxic activity.

When considering the role of disulfide bonds in toxin delivery, there are no studies addressing this question. However, the importance and function of disulfide bonds in the structural stability and activation of the catalytic activity of these proteins have already been demonstrated. For example, many staphylococcal enterotoxins, such as SpeA, have been found to contain disulfide bonds, with the disulfide loop being a characteristic feature. These bonds are essential for the biological activity of the proteins, which is crucial for their binding to their target (Lee et al. 2021). In the case of T6SS effectors, a functional disulfide bond has already been described in the *Serratia marcescens* T6SS amidase Ssp2. This regulatory disulfide, formed by DsbA, activates the amidase activity of the effector once it is secreted into the prey's periplasm (Mariano et al. 2018). It has also been demonstrated that disulfide bonds play a role in the structural stability of certain components of the T6SS machinery (Qin et al. 2016).

Our results reveal a novel role for disulfide bonds in T6SS effectors. When analyzing the cargo effectors of Hcp1, no common interaction motif appears to be present. Each effector interacts with the ring formed by the Hcp1 hexamer in a specific manner, involving different Hcp1 residues located within the inner cavity. Nevertheless, three residues (T59, S115, and T122) are essential for the secretion of all four cargo effectors (Howard et al. 2021; Silverman et al. 2013; Whitney et al. 2014). These polar residues align inside the pore, suggesting the existence of a shared interaction motif among cargo effectors, although this motif has never been identified. In other organisms, secretion motifs such as MIX (Salomon 2016), FIX (Jana et al. 2019), or RIX (Kanarek et al. 2023) have been identified, but they are absent in Hcp1 cargos. The Tse1 C‐terminal region containing C148 seems crucial for its interaction with Hcp1; however, it does not share sequence similarities with other cargo effectors like Tse2‐4. Additionally, the absence of disulfide bonds in other effectors (Lu et al. 2014; Robb et al. 2016) implies that the behavior of Tse1 cannot be generalized to all H1‐T6SS cargo effectors.

**FIGURE 5:**
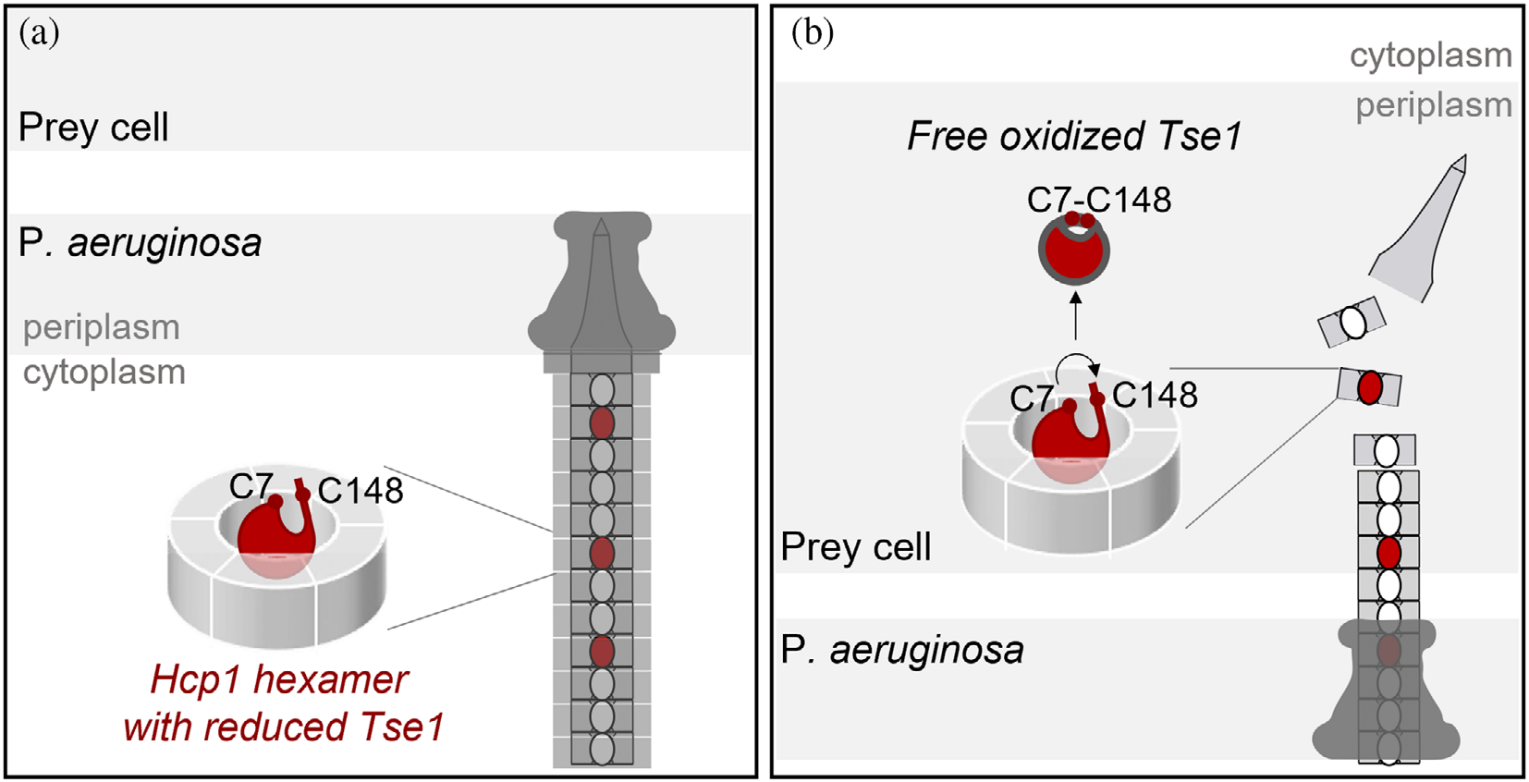
Tse1 secretion mechanism model. (a) During the assembly of Tse1 in the H1‐T6SS machinery, the internal tube of the H1‐T6SS tail is composed of Hcp1 hexamers loaded with Tse1. Tse1 is recruited in its reduced form into the Hcp1 hexamers and requires cysteine C148 to interact with Hcp1. (b) Once secreted into the prey cell, the internal tube of the H1‐T6SS disassembles to release the Tse1 effector into the periplasm. The disulfide bond between cysteines C7 and C148, which connects the N‐ and C‐terminal domains of the protein, forms in the prey's periplasm.

Our collective findings propose a new mechanism for the handling and disassembly of the H1‐T6SS cargo effector Tse1, involving a regulatory disulfide bridge (Figure 5). Our functional analysis of cysteine variants suggests that the reduced form of Tse1 interacts through its unstructured C‐terminal hydrophilic extremity, with particular emphasis on the proximal cysteine, C148, which appears to play a critical role in driving the interaction with the polar patch described within the Hcp1 pore. This interaction would facilitate Tse1's assembly within the machinery. Following secretion by the H1‐T6SS, the C7‐C148 disulfide bridge forms in the oxidizing periplasm of the prey. The formation of this bond may constrain the structural dynamics of the extremities, leading to the disassembly of the cargo effector from Hcp1, and highlighting the significant role of cysteine C7 in this process. Subsequently, Tse1 accesses its substrate and carries out its lytic function on the prey. The C7‐C148 bridge thus acts as a crucial delivery mechanism.

## 4 MATERIALS AND METHODS

### 4.1 Bacterial strains and growth media

Bacterial strains and plasmids utilized in this study are detailed in Table S2. The bacteria were cultivated aerobically at either 37 or 30°C in various growth media, including M9 minimal media, Luria‐Broth (LB), LB agar, Pseudomonas isolation agar (PIA), or synthetic minimal (SIM) (Brunet et al. 2011) media as indicated. When necessary, liquid cultures were supplemented with 1 mM IPTG. Ampicillin (Ap, 100 μg/mL), tetracycline (Tc, 15 μg/mL), gentamicin (Gm, 50 μg/mL), streptomycin (Sm, 2000 μg/mL), and kanamycin (Km, 50 μg/mL) were added to maintain plasmid selection. For killing assays, *P. aeruginosa* predator and *E. coli* prey cells were pre‐cultured overnight in 2 mL LB media with 50 μg/mL ampicillin and 30 μg/mL gentamicin, respectively, and incubated at 37°C with horizontal shaking at 180 rpm. *Pseudomonas aeruginosa* overnight cultures were diluted at a 1/25 ratio in 10 mL of fresh LB media and cultivated at 37°C with horizontal shaking at 180 rpm, while prey cells' overnight cultures were diluted at a 1/50 ratio in 10 mL of fresh SIM media supplemented with 0.02 mM IPTG and incubated at 37°C with circular shaking at 180 rpm.

### 4.2 Cloning, expression, and purification of Tse1 and its variants

The PA1844 gene encoding the Tse1 toxin was cloned into the pET22b vector using NdeI and XhoI restriction sites. Uniformly labeled protein was expressed in *Escherichia coli* BL21(DE3) strain in M9 medium supplemented with ammonium chloride (1 g/L), glucose (2 g/L), either ^13^C and ^15^N labeling or unlabeled, and ampicillin (100 μg/mL). Protein expression was induced with 0.25 mM IPTG (isopropyl β‐D‐thiogalactoside) at 20°C and allowed to continue overnight. The cells were then centrifuged, and the cell pellet was resuspended in 20 mM KPO4 buffer (pH 6) and lysed using a French Press. The recombinant protein was purified through two successive chromatography steps, Q‐sepharose FF and SP‐sepharose FF, and eluted by a NaCl gradient (50–500 mM) in 20 mM KPO4 buffer (pH 6). The protein‐rich fractions were combined and subsequently dialyzed against an appropriate buffer.

### 4.3 NMR resonance assignments

Tse1 was concentrated to 1 mM in 100 mM NaCl, 10 mM KPO_4_ buffer pH 7 supplied with 10% D_2_O, and with and without 10 mM DTT before the NMR measurements. NMR spectra were acquired on a Bruker Avance III 600 MHz spectrometer equipped with a TCI cryoprobe. Spectra were processed using Topspin (Bruker) and analyzed with Topspin or CARA software (Keller 2004). A 2D ^1^H,^15^N‐HSQC spectrum and a series of triple resonance experiments have been carried out to allow sequence‐specific backbone and side chain resonance assignments of reduced and oxidized Tse1. Backbone assignment was performed based on the set of CBCANH/CBCA(CO)NH and HNCO/HN(CA)CO experiments. Side chain resonances were assigned using HBHA(CO)NH, HBHANH, and (H)CCH‐TOCSY experiments. Resonance assignments were determined as previously described (Garcin et al. 2014). The chemical shift deviations (CSDs) for each resonance were calculated using the equation: Δ*δ*_obs_ = [Δ*δ*_HN_^2^ + (Δ*δ*_N_^2^/25)]^1/2^ where Δ*δ*_HN_ and Δ*δ*_N_ are, respectively, the proton and nitrogen chemical shift variations of each residue.

### 4.4 Determination of redox potential

For NMR spectroscopy, a sample of 0.3 mM protein was dialyzed against a 50‐mM potassium phosphate buffer at pH 7.0 containing 2 mM oxidized glutathione (GSSG). Tse1 was titrated with 200 mM reduced glutathione (GSH) in the ranges 0–30 mM. ^1^H‐^15^N HSQC spectra were recorded on a Bruker Avance III 600 MHz spectrometer equipped with a TCI cryoprobe at 298 K upon titration with reduced glutathione.

### 4.5 Assay amidase activity

The amidase activity of Tse1 was tested on “permeabilized” *E. coli* TG1 bacteria. To initiate the experiment, 30 mL of LB medium was inoculated with 300 μL of an overnight pre‐culture of *E. coli* TG1 and incubated at 37°C until the absorbance at 600 nm reached a value of 1. The cultures were then centrifuged for 10 min at 5000 rpm, and the pellet was resuspended in 7 mL of “permeabilization” buffer (TES: 40 mM Tris–HCl pH 8.0, 0.5 μM EDTA, 0.5M sucrose). Subsequently, 23 mL of TES buffer, diluted twice in water (v/v), was added, and the mixture was incubated for 30 min on ice. To induce osmotic shock, the suspension was gently mixed by inverting and rolling the tube. Five replicates of Tse1 and its variants were tested at a final concentration of 20 nM using 1 mL of the “permeabilized” bacterial mixture. Absorbance kinetics were measured at 0.5‐s intervals for 5 min, with 1 mL of the “permeabilized” bacterial mixture without enzyme serving as a control.

### 4.6 Toxicity assays in bacteria

To assess Tse1‐dependent toxicity during bacterial growth in suspension, strains of *E. coli* BL21 (DE3), *E. coli* EAEC 17.2, and *Pseudomonas aeruginosa* PAK Δ*ts* *i*1Δ*tse1* were transformed with IPTG‐inducible expression plasmids. For *E. coli* BL21 (DE3), pET22b‐based plasmids encoding the indicated proteins were used. Cultures were grown overnight in LB broth supplemented with ampicillin (100 μg/mL), washed in fresh LB, adjusted to an OD₆₀₀ of 0.02, and distributed into 96‐well plates (200 μL per well, in triplicate). The plates were incubated at 37°C with continuous shaking in a microplate reader (Tecan Spark), and IPTG was added to a final concentration of 0.1 mM after 100 min. OD₆₀₀ was measured every 4 min. For *E. coli* EAEC 17.2 and *P. aeruginosa* PAK Δ*ts*i1Δ*tse1*, pMMB67HE‐based plasmids were used for protein expression. EAEC cultures were grown in LB with ampicillin (50 μg/mL), and PAK cultures in LB with carbenicillin (500 μg/mL). Overnight cultures were diluted to an OD₆₀₀ of 0.05 in fresh LB, transferred to 96‐well plates, and monitored under the same conditions. In these strains, protein expression was induced with IPTG at a final concentration of 1 mM. OD₆₀₀ readings were recorded every 5 min.

All experiments were performed with three biological replicates and repeated independently at least three times with consistent results.

### 4.7 Construction of chromosomal variants and mutants

To generate the different chromosomal substitutions, DNA fragments corresponding to the upstream and downstream sequences (~500 bp) of the target region we want to delete codon were amplified from PAK genomic DNA using appropriate oligonucleotide pairs (Table S2). The upstream and downstream PCR products were cloned into a BamHI linearized pKNG101 suicide vector using the one step sequence and ligation‐independent cloning (SLIC) strategy (Jeong et al. 2012). To perform PAK chromosome editing, the resulting suicide plasmids were introduced into the genomic DNA through conjugative transfer by a three‐partner procedure using the *E. coli* pRK2013 strain. Substitution mutants were obtained by a double selection: first on LB agar supplemented with Irgasan (25 μg/mL) and Sm (2000 μg/mL) at 37°C, followed by NaCl‐free LB agar containing 6% sucrose at 20°C. Each mutation was checked by sequencing.

### 4.8 Killing assay

The killing assays were conducted following the protocol described by Taillefer et al. (2023). Predator cells of *P. aeruginosa* and prey cells of *E. coli* EAEC 17.2 LacZ+ were cultured to an optical density at 600 nm (OD) of 2 and 0.8, respectively. Subsequently, 1 OD unit of each strain was pelleted by centrifugation for 5 min at 3000*g*. All cell pellets were resuspended in 200 μL of LB media, without the addition of antibiotics or IPTG. The predator and prey cells were mixed in a 1:1 (v/v) ratio. For control spots containing cells alone, each cell suspension was mixed with LB media in the same 1:1 (v/v) ratio. Ten microliters of each mixture were spotted onto dry LB agar media and incubated for 4 h at 37°C. This study employed two methods for detecting bacterial competition.

#### 4.8.1 Lysis‐associated β‐galactosidase assay

Ten microliters of a 1 mM CPRG solution were dispensed onto each spot, and the killing efficiency was revealed within a few minutes. For semi‐quantitative measurements, β‐galactosidase activities were determined by measuring the intensity of CPRG absorbance using a spectrophotometer on a microplate reader (TECAN, Spark). The spots were resuspended in 1 mL of LB media, pelleted by centrifugation for 10 min at 4000*g*, and 50 μL of the supernatant was mixed with 50 μL of 1 mM CPRG. This mixture was then placed in triplicate on a black 96‐well plate. Absorbance at 572 nm was measured every minute for 10 min, and β‐galactosidase activities were expressed in μM/min using ε^M^(CPRG) = 45,000 M^−1^ cm^−1^.

#### 4.8.2 Survivors growth kinetics

Each spot was resuspended in 1 mL of LB containing 30 μg/mL gentamycin and vigorously vortexed. The suspensions were diluted at a 1/10 ratio for each killing spot, and a dilution series ranging from 10^−1^ to 10^−4^ for *E. coli* prey alone was prepared. One hundred microliters of each dilution were mixed with 100 μL of LB containing 30 μg/mL gentamycin and placed in triplicate on a clear 96‐well plate. This plate was incubated in a microplate reader (TECAN, Spark) at 30°C with shaking, and the optical density at 600 nm was measured every 5 min for 10 h. For each growth curve, the time of emergence (Te) was determined at OD of 0.6.

### 4.9 Secretion assays

*Pseudomonas aeruginosa* cells were initially cultured overnight in LB medium at 37°C. Subsequently, they were inoculated at an OD of 0.1 into 15 mL of LB medium and grown to an OD of 1 at 37°C with horizontal shaking at 180 rpm. The cultures were then centrifuged for 10 min at 4°C at 4200 rpm. Supernatants were filtered through a 0.2‐μm filter and precipitated overnight with 20% TCA at 4°C. After precipitation, the supernatants were washed twice with acetone. Both the supernatants and pellets were resuspended in loading buffer and analyzed by western blot.

### 4.10 Structural models

To investigate the relationship between Tse1, its variants, and the Hcp hexamers, all AlphaFold (Abramson et al. 2024) models were aligned to a reference ring structure. Initially, chain A of the Hcp1 monomer was reassigned based on its proximity to a reference surface residue in Tse1 (Gln76). Subsequently, the remaining Hcp1 chains were reassigned in a clockwise manner relative to the first chain, with the N‐terminus oriented toward the extracellular side of the assembled Hcp1 tube. A total of 40 AlphaFold models for Tse1 and 46 for Tse1C7S were fitted, focusing exclusively on the structure of Hcp1. Variations in the total number of models reflect the exclusion of predicted assemblies that did not match known Hcp ring structures (e.g., pentamers or helicoidal forms). Finally, pairwise RMSD calculations were performed for all models corresponding to each variant.

## AUTHOR CONTRIBUTIONS

**Marie M. Grandjean:** Writing – original draft; methodology; investigation; formal analysis; visualization. **Jean‐Pierre Duneau:** Methodology; investigation; software; formal analysis; visualization; writing – review and editing. **Edwige B. Garcin:** Investigation. **Laetitia Houot:** Visualization; writing – review and editing. **Olivier Bornet:** Investigation; methodology; resources. **Christophe Bordi:** Supervision. **Latifa Elantak:** Supervision; writing – review and editing; validation; resources. **Corinne Sebban‐Kreuzer:** Conceptualization; methodology; investigation; project administration; writing – review and editing; funding acquisition; supervision; validation; visualization; data curation; formal analysis.

## Supporting information


**Figure S1.** (a, b) ^15^N‐HSQC spectrum of the reduced (a) and oxidized (b) Tse1 in 100 mM NaCl, 10 mM KPO_4_ buffer pH 7, with and without 10 mM DTT, 10% D_2_O, at 300 K on a Bruker Avance III 600 MHz spectrometer The backbone ^1^H,^15^N correlations are labeled according to the sequence. Side chain amine resonances are indicated with gray labels for Gln and Trp residues, and with gray star for Arg residues. Side chain resonances of Gln residues are connected by horizontal lines.
**Figure S2.** Chemical shift deviations (CSDs) in the ^1^H‐^15^N resonances between reduced and oxidized Tse1 states.
**Figure S3.** NMR analysis of Tse1 variants. (a) Overlay of ^1^H,^15^N‐HSQC spectra of Tse1 in its reduced state (red), and variants C7S (orange) and C148S (yellow), recorded at 300 K on a Bruker Avance III 600 MHz spectrometer. Samples were prepared in 100 mM NaCl, 10 mM KPO4 buffer at pH 7, both with and without 10 mM DTT, and 10% D2O. (b) Chemical shift deviations (CSDs) displayed for ^1^H‐^15^N resonances between reduced Tse1 and C7S (top) and between reduced Tse1 and C148S (bottom).
**Figure S4.** Toxicity assays in bacteria. (a) Growth of *E. coli* BL21(DE3) carrying pET22b plasmids for IPTG‐inducible expression of the indicated proteins, fused or not to an N‐terminal PelB signal peptide. Cultures were grown in LB + ampicillin, and OD₆₀₀ was monitored. IPTG (0.1 mM) was added at 100 min (arrow). Cytoplasmic Tse1 served as a control. (b) Growth of *P. aeruginosa* PAKΔ*retS*Δ*tsi1tse1* carrying pMMB67HE plasmids for IPTG‐inducible expression of the indicated proteins, also with or without PelB. Cultures were grown in LB + carbenicillin, and IPTG (1 mM) was added at 150 min (arrow). Cytoplasmic Tse1 and empty vector were used as controls. Data are presented as mean ± SD; *n* = 3 biological replicates. Experiments were independently repeated four times with consistent results.

**Table S1.** Analysis of the deviation of the Tse1 atomic positions in the AlphaFold models.
**Table S2.** Bacterial strains, plasmids and oligonucleotides used in this study.

## Data Availability

The data that support the findings of this study are available from the corresponding author upon reasonable request.
