# Supplementary material for: Two cysteines control Tse1 secretion by H1‐T6SS in Pseudomonas aeruginosa
Source: Protein Sci. 2025 Jul 28;34(8):e70226. doi: 10.1002/pro.70226 (PMC12302283; doi:10.1002/pro.70226)
Supplement: Supplementary file 1 — Figure S1. (a, b) 15N‐HSQC spectrum of the reduced (a) and oxidized (b) Tse1 in 100 mM NaCl, 10 mM KPO4 buffer pH 7, with and without 10 mM DTT, 10% D2O, at 300 K on a Bruker Avance III 600 MHz spectrometer The backbone 1H,15N correlations are labeled according to the sequence. Side chain amine resonances are indicated with gray labels for Gln and Trp residues, and with gray star for Arg residues. Side chain resonances of Gln residues are connected by horizontal lines. Figure S2. Chemical shift deviations (CSDs) in the 1H‐15N resonances between reduced and oxidized Tse1 states. Figure S3. NMR analysis of Tse1 variants. (a) Overlay of 1H,15N‐HSQC spectra of Tse1 in its reduced state (red), and variants C7S (orange) and C148S (yellow), recorded at 300 K on a Bruker Avance III 600 MHz spectrometer. Samples were prepared in 100 mM NaCl, 10 mM KPO4 buffer at pH 7, both with and without 10 mM DTT, and 10% D2O. (b) Chemical shift deviations (CSDs) displayed for 1H‐15N resonances between reduced Tse1 and C7S (top) and between reduced Tse1 and C148S (bottom). Figure S4. Toxicity assays in bacteria. (a) Growth of E. coli BL21(DE3) carrying pET22b plasmids for IPTG‐inducible expression of the indicated proteins, fused or not to an N‐terminal PelB signal peptide. Cultures were grown in LB + ampicillin, and OD₆₀₀ was monitored. IPTG (0.1 mM) was added at 100 min (arrow). Cytoplasmic Tse1 served as a control. (b) Growth of P. aeruginosa PAKΔretSΔtsi1tse1 carrying pMMB67HE plasmids for IPTG‐inducible expression of the indicated proteins, also with or without PelB. Cultures were grown in LB + carbenicillin, and IPTG (1 mM) was added at 150 min (arrow). Cytoplasmic Tse1 and empty vector were used as controls. Data are presented as mean ± SD; n = 3 biological replicates. Experiments were independently repeated four times with consistent results. [file PRO-34-e70226-s001.docx]

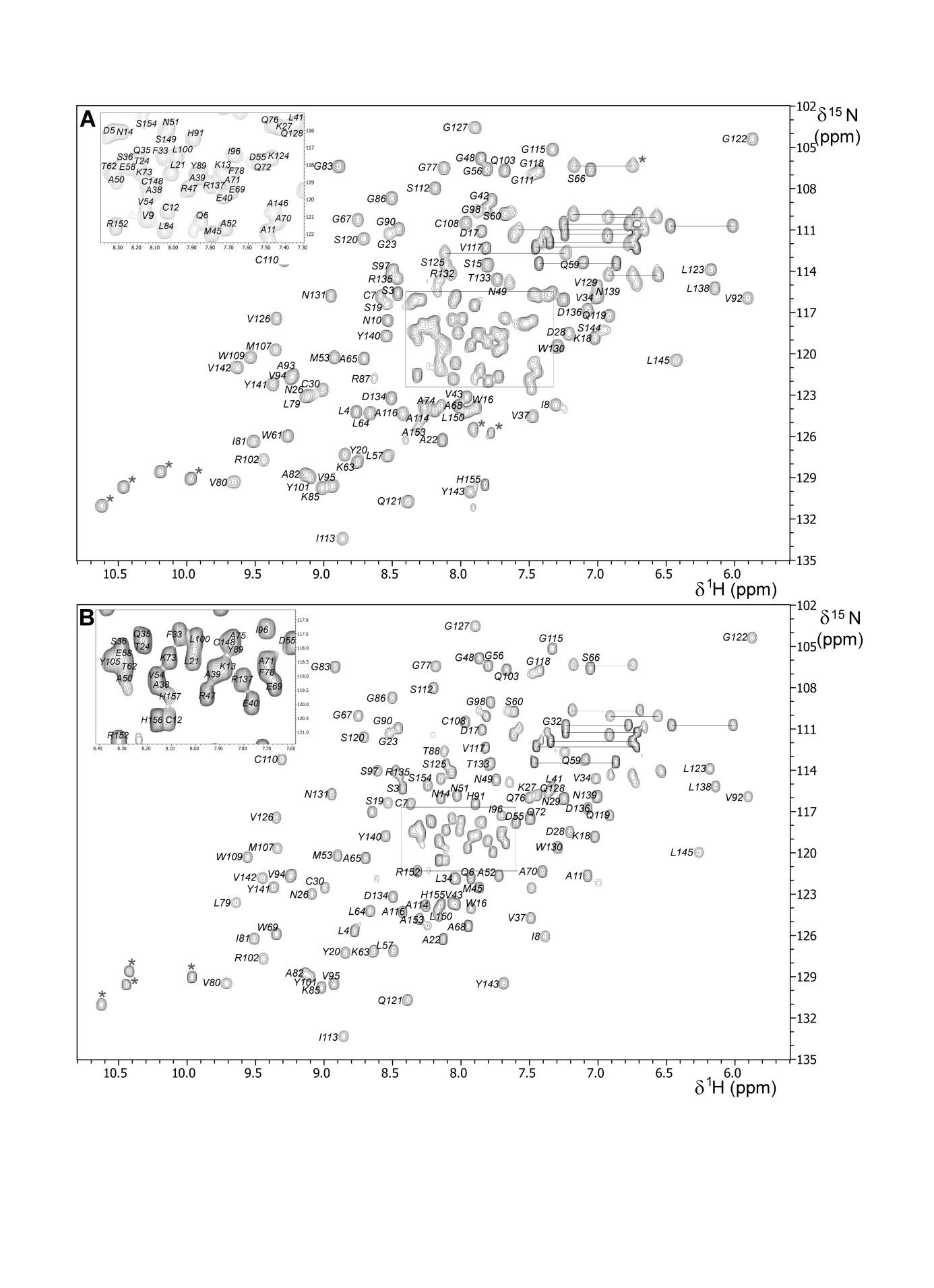


**Figures S1 : A-B. ^15^N-HSQC spectrum of the reduced (A) and oxidized (B) Tse1** in 100 mM NaCl, 10 mM KPO_4_ buffer pH7, with and without 10 mM DTT, 10 % D_2_O, at 300 K on a Bruker Avance III 600 MHz spectrometer The backbone ^1^H,^15^N correlations are labeled according to the sequence. Side chain amine resonances are indicated with grey labels for Gln and Trp residues, and with grey star for Arg residues. Side chain resonances of Gln residues are connected by horizontal lines


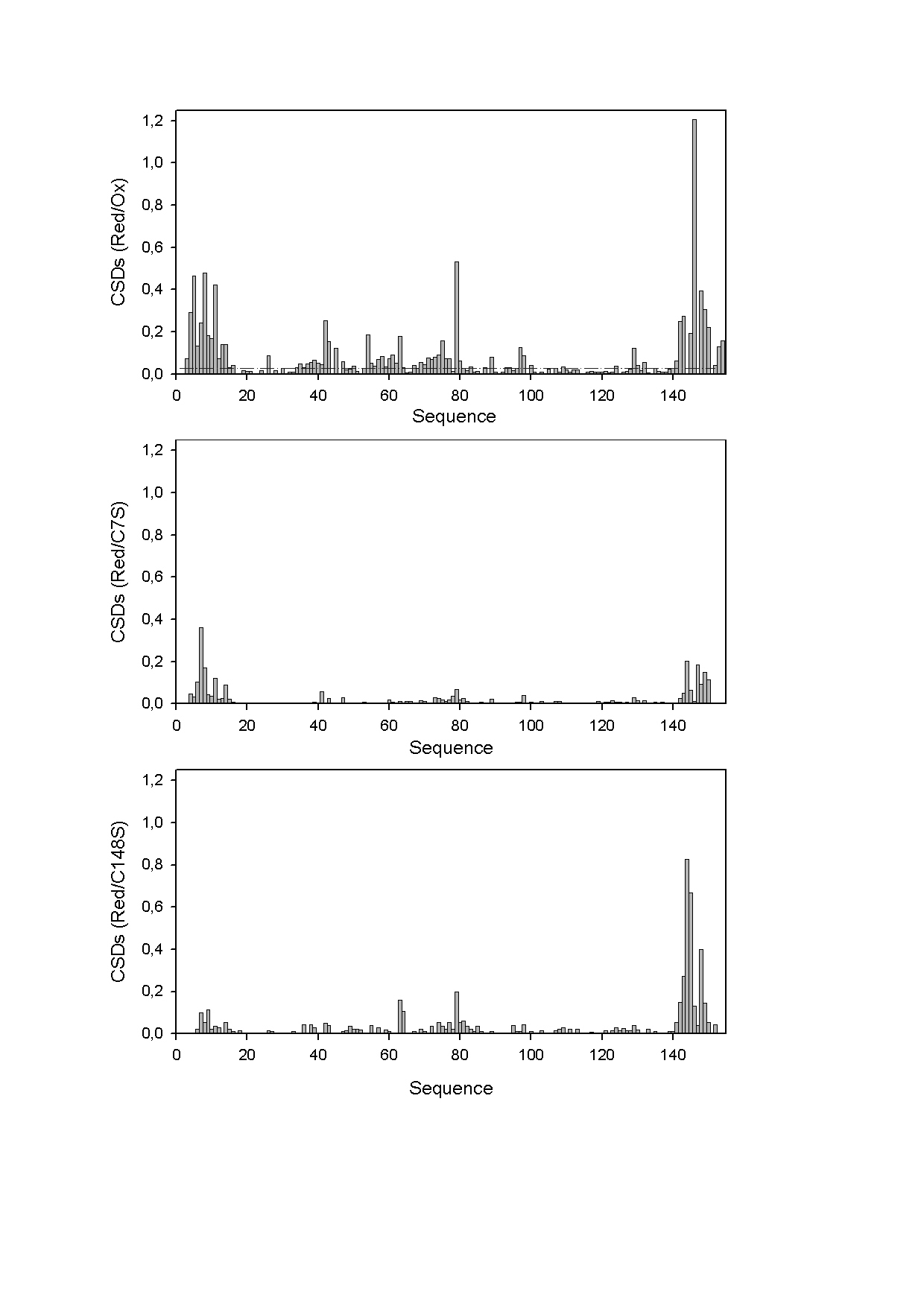


**Figure S2:** Chemical Shift Deviations (CSDs) in the ^1^H-^15^N resonances between reduced and oxidized Tse1 states


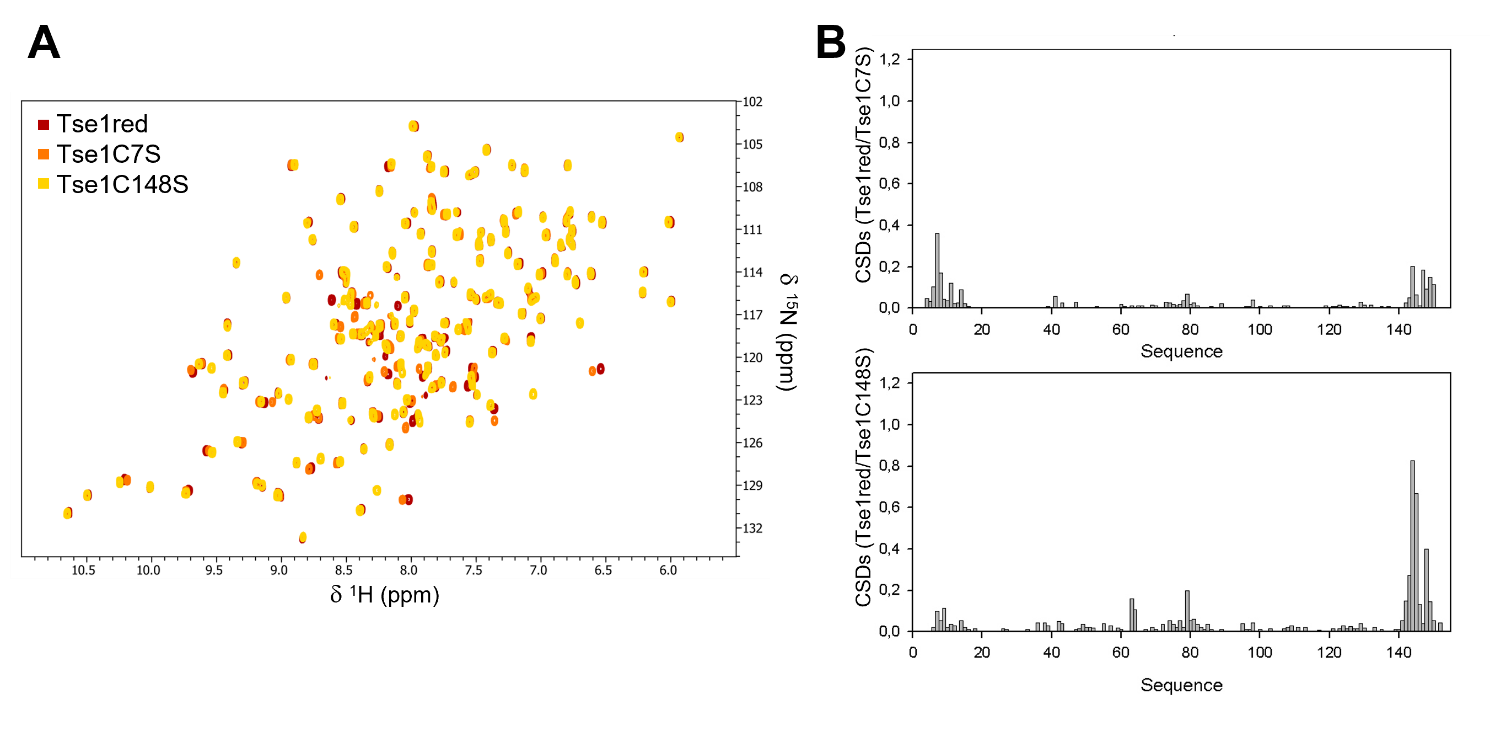


**Figure S3: NMR Analysis of Tse1 Variants A.** Overlay of ^1^H,^15^N-HSQC spectra of Tse1 in its reduced state (red), and variants C7S (orange) and C148S (yellow), recorded at 300 K on a Bruker Avance III 600 MHz spectrometer. Samples were prepared in 100 mM NaCl, 10 mM KPO4 buffer at pH 7, both with and without 10 mM DTT, and 10% D2O. **B.** Chemical Shift Deviations (CSDs) displayed for ^1^H-^15^N resonances between reduced Tse1 and C7S (top) and between reduced Tse1 and C148S (bottom).

**
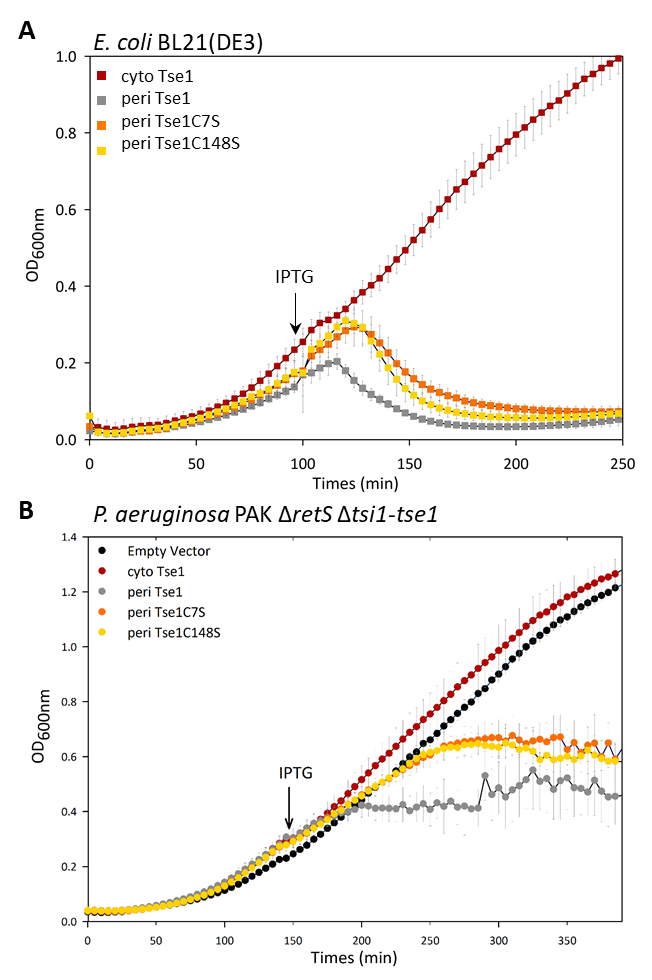
**

**Figure S4: Toxicity assays in bacteria A.** Growth of *E. coli* BL21(DE3) carrying pET22b plasmids for IPTG-inducible expression of the indicated proteins, fused or not to an N-terminal PelB signal peptide. Cultures were grown in LB + ampicillin, and OD₆₀₀ was monitored. IPTG (0.1 mM) was added at 100 min (arrow). Cytoplasmic Tse1 served as a control. **B.** Growth of *P. aeruginosa* PAKΔ*retS*Δ*tsi1tse1* carrying pMMB67HE plasmids for IPTG-inducible expression of the indicated proteins, also with or without PelB. Cultures were grown in LB + carbenicillin, and IPTG (1 mM) was added at 150 min (arrow). Cytoplasmic Tse1 and empty vector were used as controls. Data are presented as mean ± SD; n = 3 biological replicates. Experiments were independently repeated four times with consistent results.
