# Supplementary material for: Two cysteines control Tse1 secretion by H1‐T6SS in Pseudomonas aeruginosa
Source: Protein Sci. 2025 Jul 28;34(8):e70226. doi: 10.1002/pro.70226 (PMC12302283; doi:10.1002/pro.70226)
Supplement: Supplementary file 2 — Table S1. Analysis of the deviation of the Tse1 atomic positions in the AlphaFold models. Table S2. Bacterial strains, plasmids and oligonucleotides used in this study. [file PRO-34-e70226-s002.docx]

**Supplementary data**

**Table S1:** Analysis of the deviation of the Tse1 atomic positions in the AlphaFold models

|  | Average (Å) | Standard Deviation (Å) | Max (Å) | N |
| --- | --- | --- | --- | --- |
| Tse1 in | 10.1 | 3.9 | 22.5 | 24 |
| Tse1 out | 11.4 | 3.9 | 24.8 | 16 |
| C7S in | 2.1 | 0.9 | 7.6 | 36 |
| C7S out | 4.3 | 0.9 | 9.4 | 10 |

**Table S2:** Bacterial strains, plasmids and oligonucleotides used in this study

| ***E. coli* strains** | **Relevant characteristics** | **Reference** |
| --- | --- | --- |
| DH5α | *fhuA2 lac(del)U169 phoA glnV44 Φ80' lacZ(del)M15 gyrA96 recA1 relA1 endA1 thi-1 hsdR17* | Lab collection |
| TG1 | K-12 *supE thi-1 Δ(lac-proAB) Δ(mcrB-hsdSM)5, (rK-mK-)* | Lab collection |
| BL21(DE3) | *B dcm ompT hsdS*(r_B_^-^m_B_^-^) *gal* |  |
| CC118(λpir) | Host strain for pKNG101 replication, Δ(*ara-leu*) *araD* Δl*ac*X74 *galE* *galK phoA20 thi-1 rpsE rpoB* *argE*(Am) *recA1* Rf^R^ (λpir) | Lab collection |
| Sm10 | *thi-1 thr leu tonA lacY supE recA::RP4-2-Tc::Mu (*Km*^R^) supE44* | Lab collection |
| EAEC 17-2 | *Wild-type enteroaggregative Escherichia coli* | Lab collection |
| ***P.* *aeruginosa* strains** | **Relevant characteristics** | **Reference** |
| PAK | Wild type | Lab collection |
| PAK ∆*retS* | PAK strain with a deletion of the *retS* gene (PA4856) | Lab collection |
| PAK ∆*retS*∆*H1-T6SS* | PAK ∆*retS* strain with a deletion of the *H1-T6SS* gene cluster-(PA0070-PA0095) | Hachani *et al.,* 2011^63^ |
| PAK ∆*retS*∆*tsi1-tse1* | PAK ∆*retS* strain with a deletion of the tsi1 -tse1 gene (PA1844-PA1845) | Russell *et al.,* 2012^64^ |
| PAK ∆*retS* *tse1C7S* | PAK ∆*retS* strain carrying the *tse1* gene with the C7S substitution | This study |
| PAK ∆*retS* *tse1C148S* | PAK ∆*retS* strain carrying the *tse1* gene with the C148S substitution | This study |
| PAK ∆*retS*∆*pppA* | PAK ∆*retS* strain with a deletion of the *pppA* gene (PA0075) | Lab collection |
| PAK ∆*retS*∆*pppA* *tse1_6H_* | PAK ∆*retS* strain with a tag encoding 6 His residues fused to the 3' end of the *tse1* gene. | This study |
| PAK ∆*retS*∆*pppA* *tse1C7S_6H_* | PAK ∆retS strain with a 6 His tag fused to the 3' end of *tse1* carrying the C7S substitution. | This study |
| PAK ∆*retS*∆*pppA* *tse1C148S_6H_* | PAK ∆retS strain with a 6 His tag fused to the 3' end of *tse1* carrying the C148S substitution. | This study |
|  |  |  |
| **Plasmids** | **Relevant characteristics** | **Reference** |
| pMMB67HE-*tse1* | pMMB67HE vector carrying the coding sequence of the tse1 gene, inserted using XbaI and EcoRI sites, Amp^R^ | This study |
| pMMB67HE-*pelB tse1* | pMMB67HE vector carrying the coding sequence of the tse1 gene with pelB fusion, inserted using XbaI and EcoRI sites, Amp^R^ | This study |
| pMMB67HE-*pelB tse1C7S* | pMMB67HE vector carrying the coding sequence of the tse1 gene with pelB fusion and the C7S substitution, inserted using XbaI and EcoRI sites, Amp^R^ | This study |
| pMMB67HE-*pelBtse1C148S* | pMMB67HE vector carrying the coding sequence of the tse1 gene with pelB fusion and the C148S substitution, inserted using XbaI and EcoRI sites, Amp^R^ | This study |
| pET22b-*tse1* | pET22b vector carrying the coding sequence of the tse1 gene, inserted using NdeI and XhoI sites, Amp^R^ | This study |
| pET22b-*tse1C7S* | pET22b vector carrying the coding sequence of the tse1 gene with the C7S substitution, inserted using NdeI and XhoI sites, Amp^R^ | This study |
| pET22b-*tse1C148S* | pET22b vector carrying the coding sequence of the tse1 gene with the C148S substitution, inserted using NdeI and XhoI sites, Amp^R^ | This study |
| pET22b-*pelB tse1* | pET22b vector carrying the coding sequence of the tse1 gene, inserted using the SLIC method, Amp^R^ | This study |
| pET22b-*pelB tse1C7S* | pET22b vector carrying the coding sequence of the tse1 gene with the C7S substitution, inserted using the SLIC method, Amp^R^ | This study |
| pET22b-*pelBtse1C148S* | pET22b vector carrying the coding sequence of the tse1 gene with the C148S substitution, inserted using the SLIC method, Amp^R^ | This study |
| pKNG101 ∆*H1-T6SS* | Suicide vector for *H1-T6SS* gene cluster deletion by allellic replacement, Sm^R^ | This study |
| pKNG101 ∆*tsi1-tse1* | Suicide vector for *tsi1-tse1* gene deletion by allellic replacement, Sm^R^ | This study |
| pKNG101 *∆pppA* | Suicide vector for *pppA* gene cluster deletion by allellic replacement, Sm^R^ | Lab collection |
| pKNG101 *tse1C7S* | Suicide vector for *tse1-* C7S subtitution by allellic replacement, Sm^R^ | This study |
| pKNG101 *tse1C148S* | Suicide vector for *tse1-* C148S subtitution by allellic replacement, Sm^R^ | This study |
| pKNG101 *tse1_6H_* | Suicide vector for *tse1-* 6his tag insertion by allellic replacement, Sm^R^ | This study |
| pKNG101 *tse1C7S_6H_* | Suicide vector for *tse1-* C7S subtitution and 6his tag insertion by allellic replacement, Sm^R^ | This study |
| pKNG101 *tse1C148S_6H_* | Suicide vector for *tse1-* C148S subtitution and 6his tag insertion by allellic replacement, Sm^R^ | This study |
| pRK2013 | *Tra+ Mob+* Km^R^ *;* helper plasmid for triparental mating | Lab collection |
| pKNG101 | Suicide vector in *P. aeruginosa*, *sacB^+^*, Sm^R^ | Lab collection |
|  |  |  |
| **Oligonucleotides** | **Sequence (5’→3’)** | **Target** |
| ***pKNG101 ∆H1-T6SS*** |  |  |
| *∆H1-T6SS*-1 | GATTACGCGTTAACCCGGGCCCATGGTCAACGACATGGAGCTGGAG | PAK genome |
| *∆H1-T6SS*-2 | ccaAGGCCTGATC | PAK genome |
| *∆H1-T6SS*-3 | CGATCAGGCCTTGGCCAGAACTGAAGCGGCGC | PAK genome |
| *∆H1-T6SS*-4 | GGACTATAGACTATACTAGTGGTGGCGTTCAACAGTTCCATGTC | PAK genome |
| ***pKNG101-∆tsi1-tse1*** |  |  |
| ∆*tsi1-tse1*-1 | ccccctgcagGTCGACGGATCCCGTGGCAGGCGACGCATTTG | PAK genome |
| ∆*tsi1-tse1*-2 | CGGCCTGAATCACATGGGGCGGGTTCtccgttgg | PAK genome |
| ∆*tsi1-tse1*-3 | GAACCCGCCCCATGTGATTCAGGCCGTGCTGCGAATGAGCC | PAK genome |
| ∆*tsi1-tse1*-4 | CTACTTATGGTACCCGGGGATCCTGGATCACGTCCGGGCGGCTCAGC | PAK genome |
| ***pMMB67HE-tse1*** |  |  |
| *T7* | TAATACGACTCACTATAGGG | *pET22b-tse1* |
| *3'EcoRI-pet22* | ccgGAATTCTTTGTTAGCAGCCGG |  |
| *pMMB-5’* | acggcgtttcacttctgagttc |  |
| *pMMB-3’* | agcggataacaatttcacacagga |  |
| ***pET22b-tse1*** |  |  |
| *pet-tse1*-1 | CGCAACATATGGACAGTCTCGATCAATGCATCGTC | PAK genome |
| *pet-tse1*-2 | CCGCTCGAGACTGGCCCTGGGCAGGCTGCAAC | PAK genome |
| *pet-tse1C7S*-1 | GCAACATATGGACAGTCTCGATCAAAGCATCGTCAACGCC | *pET22b-tse1* |
| *pet-tse1C148S*-2 | GTGCTCGAGTCAACTGGCCCTGGGCAGGCTGCTACTGGC | *pET22b-tse1* |
| *pet-pelB-tse1*-1 | CTGCCCAGCCGGCGATGGCCatggacagtctcgatc | *pET22b-tse1* |
| *pet-pelB-tse1*-2 | gaatcaGTGGTGGTGGTGGTGGTGactggccctgggcagg | *pET22b-tse1* |
| ***pKNG101-tse1*** |  |  |
| *tse1*-1 | ccctgcaggtcgacggatccgggccggagcgccccttatc | PAK |
| *tse1_6H_* -2 | gaatcaGTGGTGGTGGTGGTGGTGactggccctgggcagg | PAK genome |
| *tse1_6H_* -3 | CACCACCACCACCACCACtgattcaggccgtgctgcgaatg | PAK genome |
| *tse1*-4 | CTTATGGTACCCGGGGATCCcgatggcctggatcacgtccg | PAK genome |
| *tse1C7S*-1 | gattacgcgttaacccgggcccCGCCGCTCTCTTCCTGAGCC | PAK or PAK tse1_6H_ genome |
| *tse1C7S*-2 | GCGTTGACGATTGATTGATCGagactgtcc | PAK or PAK tse1_6H_ genome |
| *tse1C7S*-3 | CGATCAATCAATCGTCAACGCCTGC | PAK or PAK tse1_6H_ genome |
| *tse1C7S*-4 | ggactatagactatactagtAATGCGGAGGTCGGAGTGCG | PAK or PAK tse1_6H_ genome |
| *tse1C148S*-1 | GATTACGCGTTAACCCGGGCCCGCTCGCGCCGTTCTGCCAGACC | PAK or PAK tse1_6H_ genome |
| *tse1C148S*-2 | GGGCAGGCTCGAACTGGCCAGGGAGTAG | PAK or PAK tse1_6H_ genome |
| *tse1C148S*-3 | CTGGCCAGTTCGAGCCTGCCCAGGGCC | PAK or PAK tse1_6H_ genome |
| *tse1C148S*-4 | GGACTATAGACTATACTAGTGGATCACGTCCGGGCGGCTCAGC | PAK or PAK tse1_6H_ genome |
|  |  |  |

^*^Sm^R^, streptomycin resistance; Amp^R^, ampicillin resistance; Gm^R^ gentamicin resistance; Tc^R^ tetracycline resistance.
